# A Reconfigurable Digital Beamformer Implemented on a Field-Programmable Gate Array for Real-Time and Resource-Efficient Direction-of-Arrival Estimation

**DOI:** 10.3390/s25082497

**Published:** 2025-04-16

**Authors:** Yuting Wang, Liyuan Zhu, Tianxiang Wu, Shunli Ma

**Affiliations:** 1 State Key Laboratory of Integrated Chips and Systems, College of Integrated Circuits and Micro-Nano Electronics, Fudan University, Shanghai 200433, China; 22212020033@m.fudan.edu.cn (Y.W.); liyuanzhu@fudan.edu.cn (L.Z.); 2 College of Information, Mechanical and Electrical Engineering, Shanghai Normal University, Shanghai 200234, China; 3 Shaoxin Laboratory, Shaoxing 312000, China

**Keywords:** direction of arrival (DOA), digital beamforming, FPGA, phased array

## Abstract

A medium- or large-scale receiving antenna array using digital beamforming can achieve high-resolution direction-of-arrival (DOA) estimation at the receiver. However, it typically suffers from high cost and complexity. This paper proposes an efficient reconfigurable digital beamformer that can achieve real-time angle estimation with high accuracy while making effective use of hardware resources. The digital beamformer operates in two modes: beamforming mode and angle estimation mode. In the angle estimation mode, the phase shift steps required for beam scanning can be flexibly adjusted according to the desired angular resolution. By dynamically switching operational modes and fine-tuning the granularity of processing tasks, this architecture maximizes the efficient use of Field-Programmable Gate Array (FPGA) resources, ensuring optimal performance and flexibility in real-time signal processing applications. Simulation results show that with an input signal-to-noise ratio of 10 dB, the beamformer can complete DOA estimation with an error of less than 1° within microsecond-level delay.

## 1. Introduction

Since the early 1950s, phased array technology has continuously evolved, becoming the cornerstone of modern remote sensing, communications, and electronic warfare systems [1]. In signal reception, a phased array sums the signals at the array level using electronic equipment, while in transmission, it forms a beam via radiation. With the increasing number of 5G networks and communication devices, improving communication efficiency has become particularly important [2,3,4].

Massive MIMO (Multiple Input Multiple Output) is a technology that can effectively enhance communication capacity and signal quality, while adaptive beamforming is a commonly used method to improve array performance. Paper [5] proposes a massive array adaptive beamforming method based on FPGA, combining the LMS algorithm with an efficient hardware architecture for FPGA. In addition, the growing demand for high data rate communication continues to drive the need for broadband communication systems [6]. Wideband digital beamforming (Wideband DBF) provides a high-performance solution through spatial filtering while minimizing the increase in system size. Paper [7] implements wideband digital beamforming using multi-beam time delay (MTD) elements based on Lagrange interpolation.

DOA (direction-of-arrival) estimation is a key task in massive MIMO systems [8,9,10]. This technology is based on the electromagnetic propagation characteristics of the signal transmitted by the device, where the direction of the signal received by the array antenna determines the phase shift between different received channels [11]. Various algorithms have been designed for DOA estimation [11,12,13,14,15,16,17,18,19,20,21,22,23,24,25]. The MUSIC algorithm is a classic high-resolution DOA estimation method [13]. However, as the array size increases, the computational burden of the MUSIC algorithm also grows. Specifically, when the number of array elements is very large, the time and resources required for computation increase significantly, making the algorithm less suitable for real-time applications [14]. In [15], the researchers focused on optimizing key steps of the MUSIC algorithm, such as eigenvalue decomposition (EVD), spectral estimation, and peak searching, thus reducing the algorithm complexity and introducing hardware-friendly designs to improve resource utilization and processing speed. Unlike the standard MUSIC algorithm, the Root-MUSIC method does not directly use the spectral peak but instead calculates the DOA precisely by using the roots of a polynomial [16].

The ESPRIT algorithm is another method for signal parameter estimation [17]. It divides the array’s received signals into two parts and applies the principle of rotational invariance to calculate the DOA of the signal sources, without the need to compute spectral peaks like MUSIC. Ref. [18] improves the accuracy and robustness of DOA estimation in complex signal environments by combining spatial smoothing techniques with the ESPRIT algorithm. Ref. [19] proposes an expandable DOA estimation processor based on the ESPRIT algorithm, specifically designed for frequency-modulated continuous wave radar systems. In multi-source DOA estimation, traditional methods significantly increase computational demands. However, FFT, as a spectral analysis tool, can quickly extract the direction information of signal sources [20]. The FFT-based DOA estimation method proposed in [21] demonstrates lower computational complexity and strong performance, particularly in coprime MIMO radar systems, addressing this challenge.

Despite significant advancements in DOA estimation algorithms over the past few decades, DOA estimation still faces multiple challenges, including computational complexity, hardware resource constraints, performance degradation in low-SNR environments, and estimation errors in multipath and coherent signal conditions. First, classical high-resolution algorithms (such as MUSIC and ESPRIT) rely on eigenvalue decomposition of the covariance matrix or subspace processing, leading to a rapid increase in computational complexity as the array size grows [15,19], making real-time applications difficult to achieve. Second, in hardware implementation, DOA estimation requires large-scale parallel computing capabilities, while FPGA or ASIC hardware platforms have certain limitations in terms of resources and power consumption [24,25,26]. Additionally, in low-SNR or high-noise environments, the separation between the noise subspace and the signal subspace becomes challenging, resulting in reduced DOA estimation accuracy. Multipath effects and signal coherence can also impact algorithm performance, causing estimation deviations or even failures [27,28,29].

To further improve DOA estimation accuracy, larger-scale arrays and higher frequency bands have been employed [30]. Millimeter waves offer advantages such as large bandwidth, high data rates, and low latency, while large-scale MIMO increases system capacity and spectral efficiency by utilizing numerous antenna arrays. By increasing the number of array elements, large antenna arrays enhance spatial resolution and beamforming capabilities, which not only improve DOA estimation accuracy but also reduce communication path loss by focusing signal energy in a specific direction. Additionally, since beamforming operates at the physical layer, it provides significant advantages in computational efficiency and power consumption compared to application-layer operations in the network protocol stack [22].

In massive MIMO systems, hybrid analog and digital architectures are often employed due to hardware complexity and power consumption limitations. For hybrid MIMO architectures, Ref. [24] proposes three hybrid subarray DOA estimation methods, focusing on reducing complexity while maintaining accuracy. Ref. [25] introduces a DOA estimation method based on hybrid antenna arrays with unequal-sized subarrays, combined with a three-stage beam-scanning strategy, which significantly improves scanning efficiency and effectively addresses direction ambiguity. The DOA estimation problem for hybrid arrays remains an open and cutting-edge issue [9].

Digital beamforming at the receiver is a powerful signal processing technique widely applied in radar and communication fields [31]. The architecture of a traditional digital beamforming receiver consists of five main components: the Radio Frequency (RF) modulation stage, an Analog-to-Digital Converter (ADC), the Digital Down Conversion (DDC) stage, the Complex Weight Multiplication (CWM) stage, and the Summation stage [32]. However, despite its many advantages, the traditional digital beamforming radar receiver architecture involves a large number of microwave and digital channels (each with multiple ADCs), making the implementation of digital beamforming costly. As a result, compared to other beamforming methods, its applications are more limited [33].

In recent years, many novel algorithms have been proposed to overcome the problem of DOA estimation in complex signal environments. For example, the digital beamforming method based on Quantum Support Vector Machine (QS-SVM) was introduced in [34], demonstrating its efficient hardware implementation capability on FPGA platforms. Additionally, the subspace deconvolution algorithm proposed in [35], particularly designed for high-resolution and robustness requirements, shows excellent performance in complex environments. The DOA estimation method based on Sparse Bayesian Learning, presented in [36], models the signal sources as a sparse process and uses Bayesian inference to handle the unknown information of the sources, effectively addressing the off-grid source estimation problem. In [37], a structured Nyquist correlation reconstruction method was proposed specifically for DOA estimation with sparse arrays. By solving the information loss problem caused by sparse arrays, this method significantly improves the accuracy and robustness of DOA estimation, especially in low-SNR and high-noise environments. Furthermore, Ref. [38] significantly improves DOA estimation accuracy in low-SNR conditions by combining Support Vector Regression (SVR) with signal correlation analysis. Most recently, a notable contribution in this area is the work of [39], which introduced a novel hybrid radar fusion model. By fusing information from different DL and UL frequency bands and leveraging multiple snapshots from diverse frequency bands, Ref. [39] proposed a fused MUSIC estimator that achieves superior DOA estimation performance in multi-band scenarios, setting a new benchmark for DOA estimation in complex environments.

This paper focuses on the hardware design of a low-complexity reconfigurable DOA estimation system using traditional beamforming methods. In this study, we assume that the proposed method can estimate only one DoA at a time. Since we focus on the Line-of-Sight (LoS) path, the Non-Line-of-Sight (NLoS) paths are treated as interference in the DoA estimation problem. The main contributions of this paper are summarized as follows:

1. A multi-channel digital beamformer with real-time DOA functionality based on traditional beam scanning is designed. The system allows for the reconfiguration of the number of phase shifts and the size of the weight memory according to the desired DOA estimation angular resolution.
2. The specific FPGA hardware implementation of the DOA system is presented, with theoretical derivation provided. The hardware platform used is a Xilinx Kintex-7 series FPGA device, specifically the XC7K70T (XC7K70TFBV676-1).
3. Simulation results on the MATLAB R2023b and VIVADO 2023.2 platforms are provided to validate the performance of the reconfigurable digital beamformer under different scanning resolutions, with comparisons made to the results from other classical DOA algorithms.

The remainder of the paper is structured as follows: Section 2 discusses the theoretical foundations of beamforming and DOA estimation; Section 3 presents the hardware architecture of the proposed DOA system; Section 4 provides simulation results and performance analysis; and finally, Section 5 concludes the paper with a summary of the findings.

## 2. Theoretical Background

**Figure 1:**
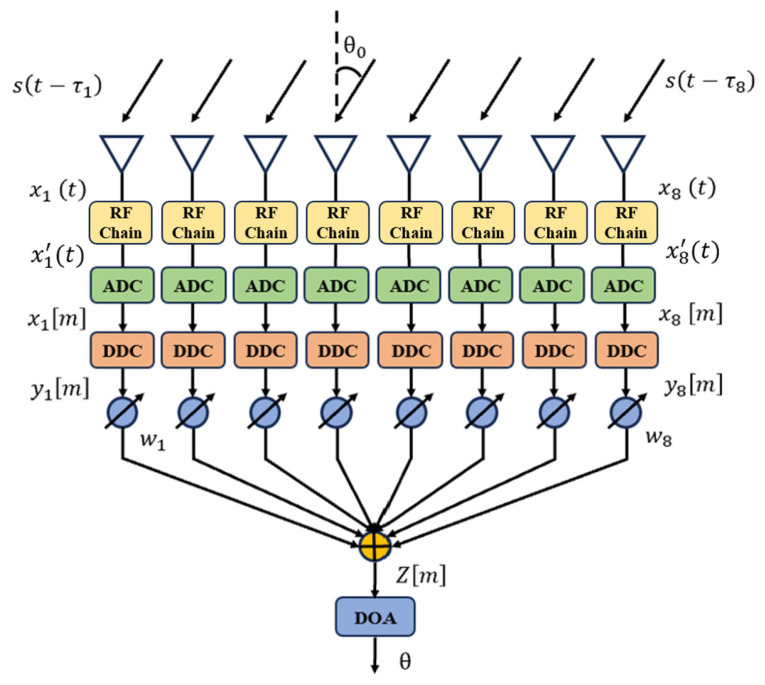
Structure of the receiver-side digital beamformer.

Figure 1 illustrates the block diagram of the digital beamformer at the receiver. It is assumed that a Uniform Linear Array (ULA) consists of N antenna elements, and the signal received by each antenna element is

(1) $$x_{n}\left(t\right)=s\left(t-\tau_{n}\right)+\eta_{n}\left(t\right)$$

where $s\left(t\right)$ is the narrowband signal, $\tau_{n}=\frac{d_{n}sin(\theta_{0})}{c}$ is the time delay of the signal arriving at the *n*-th array element, $d_{n}$ is the position of the n-th array element relative to the reference point, d is the spacing between adjacent array elements, $\theta_{0}$ is the angle of arrival, and $\eta_{n}\left(t\right)$ is the Gaussian white noise.

For narrowband signals, the time delay and phase shift can be considered interchangeable. Therefore, the RF signal received by the receiver can be expressed as

(2) $$x_{n}\left(t\right)=Acos\left(\omega_{c}t+\emptyset_{n}\right)+\eta_{n}\left(t\right)$$

where

(3) $$\emptyset_{n}=-\beta d_{n}sin\left(\theta_{0}\right)=-\frac{2\pi d_{n}sin\left(\theta_{0}\right)}{\lambda }=-\frac{2\pi \left(n-1\right)dsin\left(\theta_{0}\right)}{\lambda }$$

The above equation indicates that, for a ULA receiving array, each incident angle $\theta_{0}$ within the range $\left[-\frac{\pi }{2},\frac{\pi }{2}\right]$ corresponds uniquely to the phase difference of the sinusoidal signal. By solving the phase differences between array elements, the incident angle of the array signal can be directly determined. When *d* = *λ*/2 is chosen, Equation (3) can be rewritten as Equation (4):

(4) $$\emptyset_{n}=-\pi \left(n-1\right)sin\left(\theta_{0}\right)$$

From this formulation, it is evident that the phase difference between array elements depends solely on the angle of incidence and is independent of both the carrier frequency and the element spacing. In practical array signal processing systems, the RF signal *x*(*t*) received by the antenna is first amplified, filtered, and down-converted to an analog intermediate frequency (IF) signal *x*′(*t*) by the RF front-end. The IF signal *x*′(*t*) of the *n*-th channel can be expressed as

(5) $${x^{\prime }}_{n}\left(t\right)=Acos\left(\omega_{IF}t+\emptyset_{n}\right)+\eta_{n}\left(t\right)$$

This step reduces the processing burden on the ADC. The ADC then converts *x*′(*t*) into a digital signal, which is subsequently processed by the digital beamformer. Since the digital beamformer operates directly *x*′(*t*), and the direction-of-arrival (DOA) estimation is theoretically unaffected by the carrier frequency, this design focuses primarily on the ADC and IF signal processing stages. To simplify the model, we assume an ideal RF front-end and antenna array, with no mutual coupling between antenna elements and no additional noise or nonlinear effects introduced by the RF circuitry. For completeness, however, we assume a carrier frequency of *fc* = 1 GHz and an antenna spacing of *d* = *λ*/2.

The ADC samples the signal from each array element at the sampling frequency to obtain the digital signal:

(6) $$x\left[m\right]=x_{n}\left(mT_{S}\right)=Acos\left(\omega_{IF}mT_{S}+\phi \left[m\right]\right){+\eta }_{n}\left[m\right]$$

The digital mixer multiplies each signal with the two orthogonal signals generated by the digital local oscillator to obtain ${MIX_{I}}_{n}$ and ${MIX\_Q}_{n}$:

(7) $${MIX_{I}}_{n}\left[m\right]=Acos(\omega_{IF}mT_{S}+\phi \left[m\right])\cdot cos\left(\omega_{LO}mT_{S}\right)\;=\frac{A}{2}\left[\cos\left(\left(\omega_{IF}+\omega_{LO}\right)mT_{S}+\phi \right)+\cos\left(\left(\omega_{IF}-\omega_{LO}\right)mT_{S}+\phi \right)\right]$$

(8) $${MIX\_Q}_{n}[m]=Acos(\omega_{IF}mT_{S}+\phi \left[m\right])\cdot sin\left(\omega_{LO}mT_{S}\right)\;=\frac{A}{2}\left[\sin\left(\left(\omega_{IF}+\omega_{LO}\right)mT_{S}+\phi \right)+\sin\left(\left(\left(\omega_{IF}-\omega_{LO}\right)mT_{S}+\phi \right)\right)\right]$$

After filtering with a low-pass filter, two orthogonal signals $I_{n}$ and $Q_{n}$ are obtained:

(9) $$I_{n}\left[m\right]=\frac{A}{2}\cos\left(\left(\omega_{IF}-\omega_{LO}\right)mT_{S}+\phi \right),$$

(10) $$Q_{n}\left[m\right]=\frac{A}{2}\sin\left(\left(\omega_{IF}-\omega_{LO}\right)mT_{S}+\phi \right)$$

The baseband signal can be expressed as

(11) $$y_{n}\left[m\right]=I_{n}\left[m\right]+{jQ}_{n}\left[m\right]=\frac{A}{2}e^{j\left(\omega_{IF}-\omega_{LO}\right)mT_{S}}\cdot e^{j\phi \left[m\right]}$$

Then the baseband signals from the N array elements are weighted and summed to obtain the beamforming signal $Z\left[m\right]$:

(12) $$Z\left[m\right]=\sum_{n=0}^{N-1}w_{n}y_{n}\left[m\right]=\frac{A}{2}e^{j\left(\omega_{IF}-\omega_{LO}\right)mT_{S}}\sum_{n=0}^{N-1}{w_{n}\cdot e}^{-j\frac{2\pi \left(n-1\right)dsin\theta_{0}}{\lambda }}.$$

Since array signal processing is typically more convenient when handled in matrix form, the time-domain digital signals need to be re-expressed in matrix form. For an array with N elements, collecting M snapshots over a period results in all sampled vectors $y\left[m\right]$ being organized into an N×M signal matrix:

(13) $$Y=\left[\begin{array}{ccc} y_{1}\left[1\right] & \cdots & y_{1}\left[M\right]\\ \vdots & \ddots & \vdots \\ y_{N}\left[1\right] & \cdots & y_{N}\left[M\right] \end{array}\right]$$

Each column is an N×1 sampling vector y[m], where

(14) $${y\left[m\right]=\left[y_{1}\left[m\right],\;y_{2}\left[m\right],\ldots ,y_{N}\left[m\right]\right]}^{T}$$

The part in the formula that represents the signal’s spatial information $\sum_{i=0}^{N-1}{w_{n}\cdot e}^{-j\frac{2\pi \left(n-1\right)dsin\theta_{0}}{\lambda }}$ can also be transformed into matrix form:

(15) $$AF=\sum_{n=0}^{N-1}{w_{n}\cdot e}^{-j\beta d_{n}sin\theta_{0}}=w\cdot \alpha \left(\theta_{0}\right)$$

where AF (array factor) is referred to as the array response vector corresponding to the direction angle $\theta_{0}$, $w={\left[w_{1}\cdot \cdot \cdot \;w_{N}\right]}^{T}\;$ is the array weight vector, and $\alpha \left(\theta \right)={[e}^{-j\beta d_{1}sin\theta_{0}}\;\cdot \cdot \cdot e^{-j\beta d_{N}sin\theta_{0}}]$.

The output power of the array signal can be defined as

(16) $$P_{out}=\frac{1}{M}\sum_{m=1}^{M}w^{H}y\left[m\right]y^{H}\left[m\right]w$$

The DOA problem is equivalent to finding an appropriate w such that $P_{out}$ reaches its maximum at the target θ. The weight $w_{n}$ of the n-th channel represents a digital phase shifter, where $w_{n}$ = $e^{\emptyset_{n}}$. Essentially, the DOA problem involves determining $w_{n}$ for each channel by adjusting the phase of each channel, causing phase interference among the signals. When the output power of the received signal reaches its maximum, the phase differences $\emptyset_{n}$ between the channels are obtained. By observing (13) and (14), it can be noted that when $w_{n}=e^{\pi \left(n-1\right)sin\left(\theta_{0}\right)}$, the phases of all channels are perfectly aligned, and the array output power $P_{out}$ reaches its maximum.

The number of weights Q is related to the scanning step size of the phase shifter. If the phase shifter scans within the range of $-\frac{\pi }{2}$ to $\frac{\pi }{2}$, the number of weights Q can be defined as

(17) $$Q=\frac{\pi }{stepsize}$$

For a set of N-element signals with snapshots, calculating (14) requires $N^{2}$ multiplications and the accumulation of M fixed-point numbers, resulting in a computational complexity of $O\left(MN^{2}\right)$. If there are Q weights in total, $P_{out}$ needs to be computed Q times, making the complexity of this beam-scanning algorithm for calculating a single angle $O(QMN^{2})$. It can be observed that as the number of array elements increases, the computational complexity of DOA estimation grows quadratically.

To further reduce the computational complexity and improve the real-time processing speed of DOA estimation, a simple yet effective modification method is proposed here. By rewriting $w_{n}$ as $w_{n}[m]$, the beamforming output signal under a new weight can be obtained for each snapshot through weight scanning. Simulation results later show that this scanning method can achieve satisfactory DOA results when the signal-to-noise ratio (SNR) is greater than 0 dB. The modified array output result can be expressed as

(18) $$Z\left[m\right]=\sum_{n=0}^{N-1}w_{n}\left[m\right]y_{n}\left[m\right]=\frac{A}{2}e^{j\left(\omega_{IF}-\omega_{LO}\right)mT_{S}}\sum_{n=0}^{N-1}{w_{n}\left[m\right]\cdot e}^{-j\pi \left(n-1\right)sin\left(\theta_{0}\right)}$$

For the beamforming output Z[m] under each scanning weight, the magnitude is calculated to obtain the output power corresponding to the current scanning weight, thereby determining the DOA at the maximum output power. Since weight scanning and weighted multiplication are performed simultaneously, no additional scanning is required. This method only requires weighted multiplication for M snapshots across N channels and magnitude computation for Q points, resulting in a computational complexity of only O(MNQ), making it suitable for large-scale arrays.

## 3. Hardware Implementation

**Figure 2:**
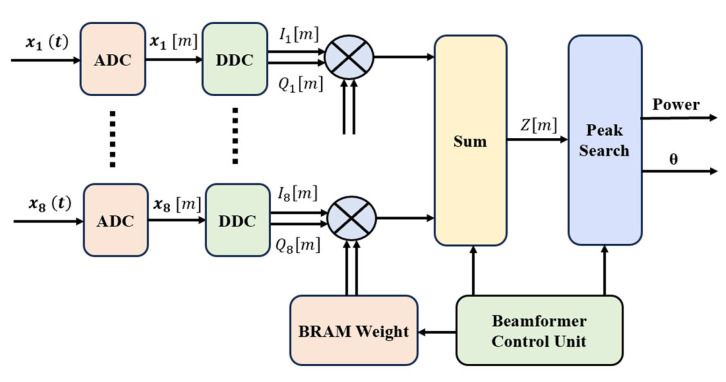
Hardware implementation of the digital beamforming system.

Figure 2 shows the hardware implementation of the digital beamforming system. This module is based on an FPGA hardware platform and consists of digital down-conversion (DDC), complex weight multiplication, weight memory, a beam control unit, and a beam-scanning module.

**Figure 3:**
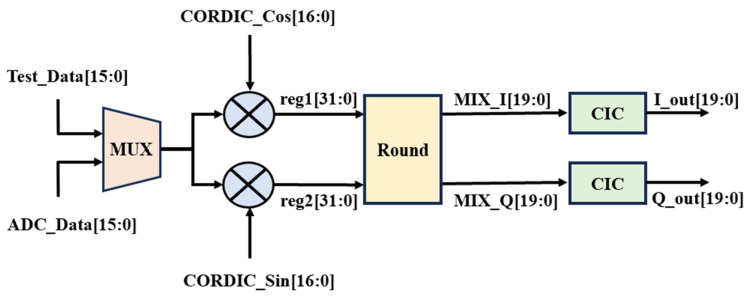
Digital down-conversion module.

Figure 3 illustrates the digital down-conversion module, which first performs digital down-conversion on the input eight-channel signals. Each channel includes digital mixing and low-pass filtering operations. The input signals are represented as 16-bit fixed-point numbers. The mixer uses a numerically controlled oscillator (NCO) to generate 17-bit fixed-point sine and cosine signals. The NCO is implemented using a structure based on the CORDIC algorithm. Each receiving channel employs two mixers to mix the input data with the orthogonal sine and cosine signals generated by the NCO, resulting in 32-bit high-frequency and low-frequency signals. To reduce computational pressure in subsequent stages, a truncation module is used to truncate the 32-bit fixed-point numbers to 20 bits. A CIC filter then removes the high-frequency components, ultimately yielding two IQ orthogonal baseband signals.

Although the truncation module helps reduce the computational and storage burden of subsequent modules, it introduces quantization error, which degrades the signal-to-noise ratio (SNR). Therefore, a quantization error analysis of the truncation module is necessary. The truncation error can be approximated as a uniformly distributed noise signal, and its mean squared error (MSE) or root-mean-square (RMS) error can be estimated using the following equation:

(19) $$Truncation\;Error=\frac{R}{\sqrt{12}}$$

where R is determined by the number of discarded bits:

(20) $$R=2^{discarded\;bits}=2^{12}=4096$$

Thus, the RMS value of the truncation error is

(21) $$RMS=\frac{4096}{\sqrt{12}}\approx 1183.72$$

The SNR of the truncated signal relative to the truncation noise can be calculated as follows:

(22) $${SNR}_{trunc}=6.02\times retained\;bits\left(dB\right)$$

Since the module retains 20 bits, the estimated SNR after truncation is

(23) $${SNR}_{trunc}=6.02\times 20=120.4\left(dB\right)$$

This indicates that truncating the signal to 20 bits does not significantly impact signal quality, as an SNR of 120.4 dB is sufficiently high to minimize quantization error effects.

**Figure 4:**
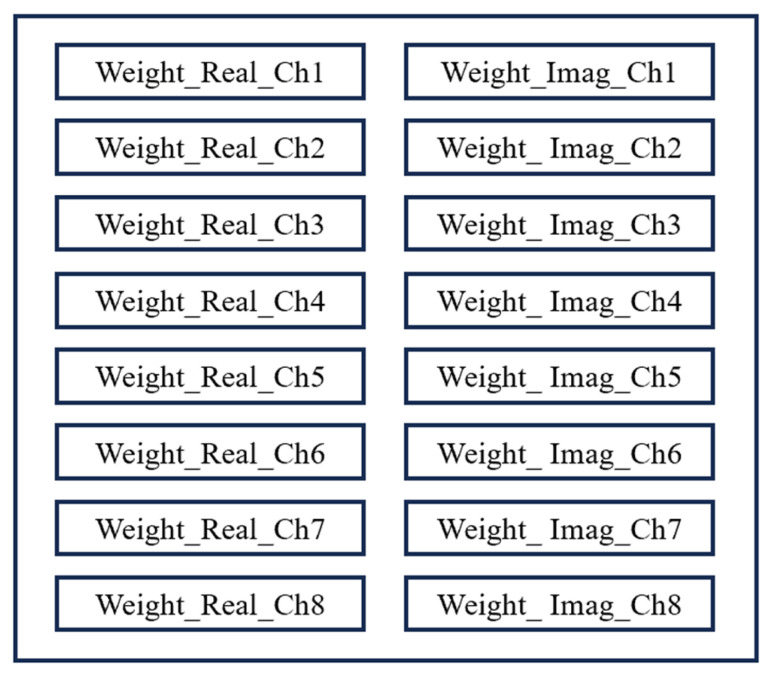
Block diagram of the weight memory.

During the beamforming weight loading stage, the module loads the real and imaginary parts of the weights separately from the Block Random Access Memory (BRAM), as shown in Figure 4. It then performs complex multiplication for each channel signal to obtain the weighted intermediate-frequency signal. The weight memory is implemented using dual-port BRAM, with separate storage units for the real and imaginary parts of each channel, supporting parallel reading and real-time updates. The bit width and depth of the BRAM can be adjusted according to the phase-shifter scanning step size. The complex multiplier adopts a pipelined structure to improve computational throughput and supports complex multiplication of 20-bit input signals with BRAM weights.

**Figure 5:**
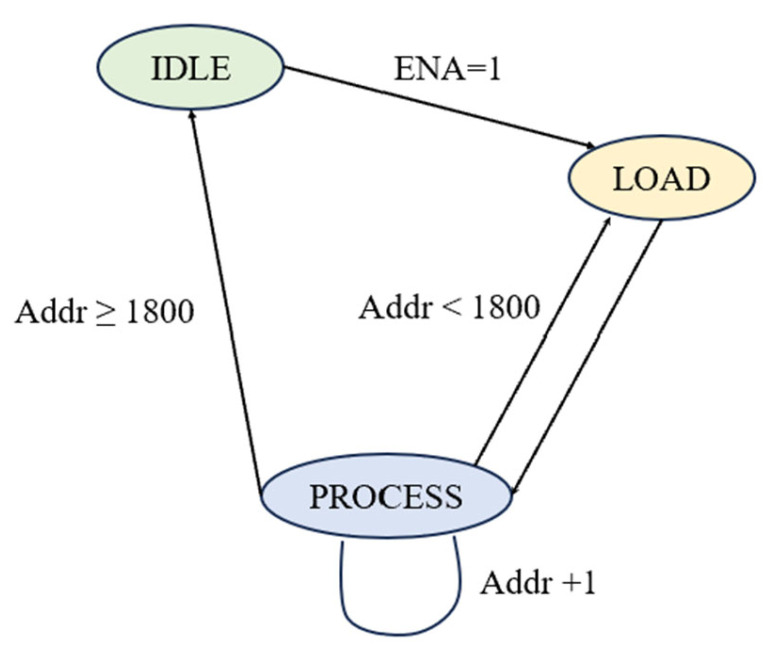
State machine diagram of the beam control unit.

The designed beam control module is a finite state machine (FSM), used for beamforming weight loading and data processing. This module controls state transitions and address generation to enable step-by-step data reading from the BRAM (weight memory) and input data loading for the complex multiplier, thereby providing an orderly data flow for subsequent beamforming processing. Figure 5 shows the state machine when the scanning step size is 0.1°, corresponding to a weight memory address depth of 1800.

**Figure 6:**
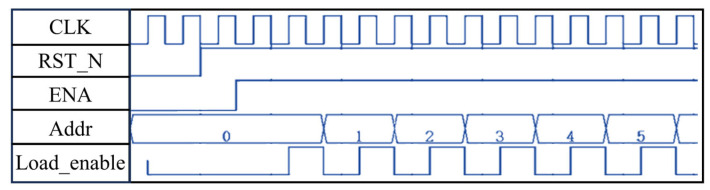
Beam control signal waveform diagram.

The state machine is triggered by the rising edge of the clock and performs state transitions based on the current state, enable signal (ENA), and address value (Addr). As shown in Figure 6, when the reset signal rst_n is set low, the state machine returns to the initial IDLE state. In the PROCESS state, the addr value increments with each clock cycle to ensure sequential reading of data from the BRAM. During reset, the address register is cleared. The load control signal load_enable is set high only in the LOAD state to control the data input for the complex multiplier.

**Figure 7:**
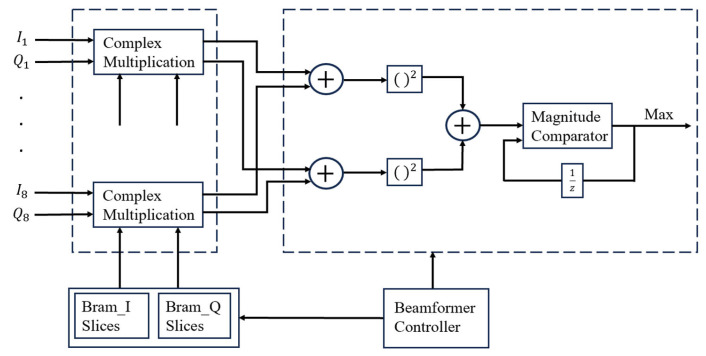
Beamforming and beam-scanning module.

Finally, as shown in Figure 7, power calculation is performed using a square accumulator, and the beam-scanning module monitors the signal strength in real time by computing the accumulated power. This module also provides a maximum power detection function and records the corresponding address information. It ultimately outputs the beamforming result for each scanning weight and records the corresponding weight address.

## 4. Approach Validation

This design uses Vivado and MATLAB software for joint simulation and verification. The hardware platform is based on the Xilinx Kintex-7 series FPGA, specifically the XC7K70T (XC7K70TFBV676-1) device. The system consists of an eight-element uniform linear array (ULA) with an element spacing of half a wavelength (d = λ/2), where the wavelength λ is calculated based on the speed of light ($c=3\times 10^{8}$ m/s) and the IF signal frequency. The sampling rate fs is set to 100 MSPS. The maximum signal amplitude is set to 32,767, corresponding to the range of a 16-bit signed integer.

### 4.1. Beamforming Mode

To verify the functionality of the beamformer, we generate multi-source incident signals using MATLAB, which include a main signal ($\theta_{1}$ = 10°) and an interference signal ($\theta_{2}$ = 25°), in order to evaluate the beamforming capability of the beamformer for multiple incident sources. The signal-to-interference ratio (SIR) between the main signal and the interference signal is 6 dB. The corresponding phase-delayed signal for each array element is constructed by calculating the phase shift of the steering vector.

#### 4.1.1. Beamforming Pattern

**Figure 8:**
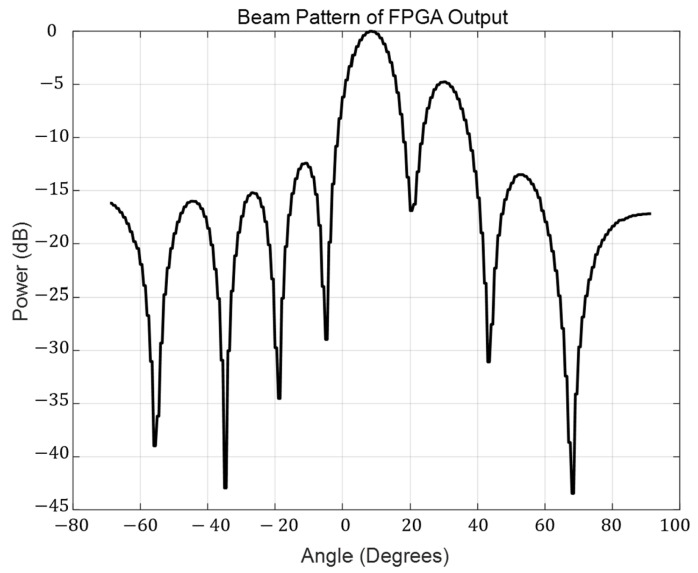
Beam pattern of FPGA output.

The simulation output waveform of the beamforming is shown in Figure 8. The array forms a main lobe at a scanning angle of 8°, with a side lobe formed next to the main lobe. It can be seen that the digital beamformer is capable of distinguishing between the main signal and the interference signal and provides an estimation of the arrival angle of the main signal. It can be observed that the beamformer forms a main lobe at 8° and a side lobe at 29°. The main lobe width, defined by the half-power beamwidth (HPBW), is 12.5°, and the main lobe width, defined by the distance between the nulls of the main lobe (FNBW), is 45.84°. The power difference between the main lobe and the side lobe is 4.8 dB.

#### 4.1.2. FFT Spectrum

**Figure 9:**
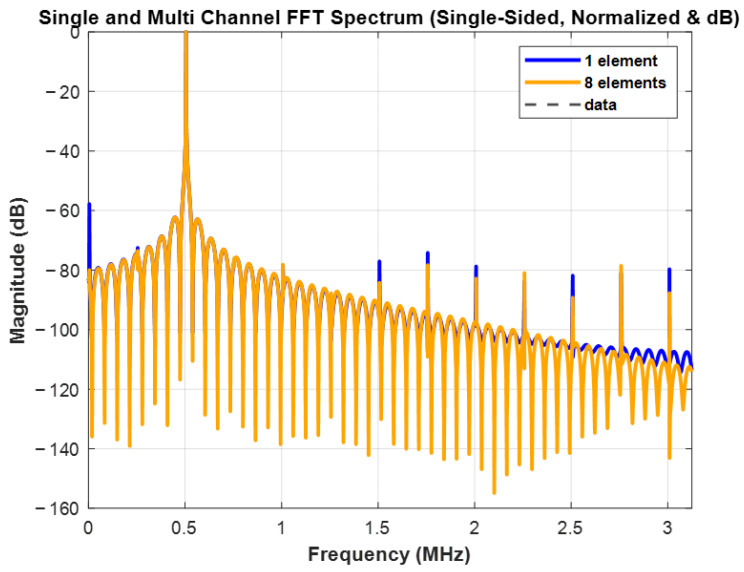
Single and multi-channel spectrum.

By fixing the complex weights of the beamformer according to the DOA detection values and performing FFT spectrum analysis on the output signals of both the single-channel and eight-channel systems, the results are shown in Figure 9. The blue curve represents the spectrum of the single channel, while the orange curve represents the spectrum of the eight channels. Both the single-channel and eight-channel systems sample 65,536 complex points, with FFT performed in MATLAB to plot the single-sided spectrum. From the figure, it can be observed that using eight channels results in higher gain in the lower frequency range compared to using a single channel. When synthesizing with eight channels, the main peak of the spectrum becomes more concentrated, the main lobe width narrows, and the gain increases. This demonstrates that using more array elements improves the system’s directionality and spectral resolution. Increasing the number of array elements enhances the system’s ability to focus on the target signal while reducing the influence of surrounding noise.

### 4.2. Angle Estimation Mode

For a given signal incident angle, the phase shift of each array element is calculated using array geometry. The generated sinusoidal signal represents the reception of a narrowband plane wave at each element of the array. The SNR range is defined from −10 dB to 30 dB. For each SNR value, Gaussian white noise is added to the signal using MATLAB’s awgn function, simulating real signal inputs under different noise conditions. The noisy signals are then quantized, converted into 16-bit integers, and saved as memory files (*.mem) in hexadecimal format. The generated .mem files can be directly used for signal processing verification on FPGA and other hardware platforms, facilitating the testing and optimization of hardware implementations.

#### 4.2.1. DOA Results

**Figure 10:**
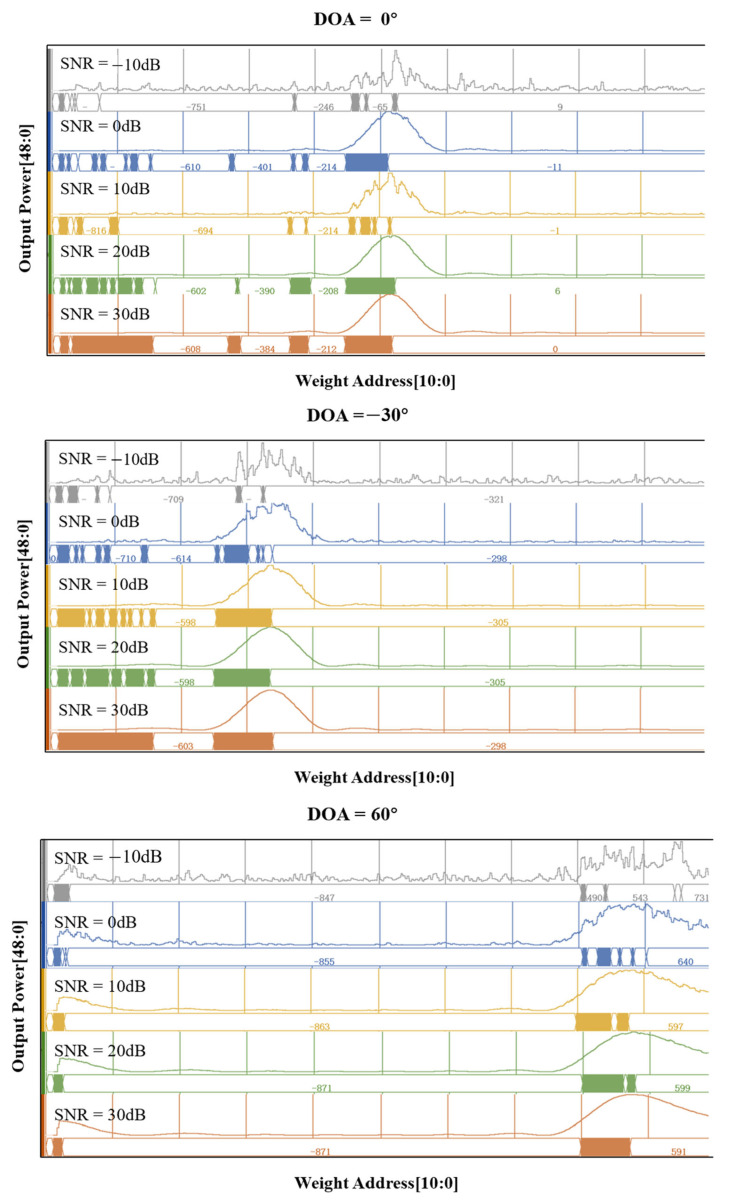
DOA output with a scan step of 0.1°.

Then, we changed the input signal’s angle and performed DOA simulation one by one in VIVADO. The FPGA clock frequency was set to 100 MHz. Figure 10 shows the output of the beamformer when the input angles are 0°, −30°, and 60°. The Output Power represents the result of beamforming the eight-channel signals with different weights, expressed as a 49-bit fixed-point number with the highest bit as the sign bit. The Weight Address is the address of the weight, expressed as an 11-bit fixed-point number. Due to linear scanning, each address directly corresponds to a weight angle. In the simulation, we verified two cases with scan steps of 1° and 0.1°.

The following figure shows the DOA results for input angles of 0°, −30°, and 60° with SNR values of −10 dB, 0 dB, 10 dB, 20 dB, and 30 dB. It can be observed that when SNR ≤ 0 dB, there is significant noise in the beamforming result. When SNR ≥ 10 dB, the beamformer can form a main lobe at the incident angle and provide angle information. Since the scan is pre-stored in the FPGA’s BRAM, the resolution of the weight scan can be adjusted. According to the simulation results, when the resolution is 0.1°, the delay is less than 40 μs, and when the resolution is 1°, the delay is less than 4 μs. Table 1 summarizes the DOA results when the scanning step is 0.1°.

#### 4.2.2. Input-Output Characteristic Curve of DOA

**Figure 11:**
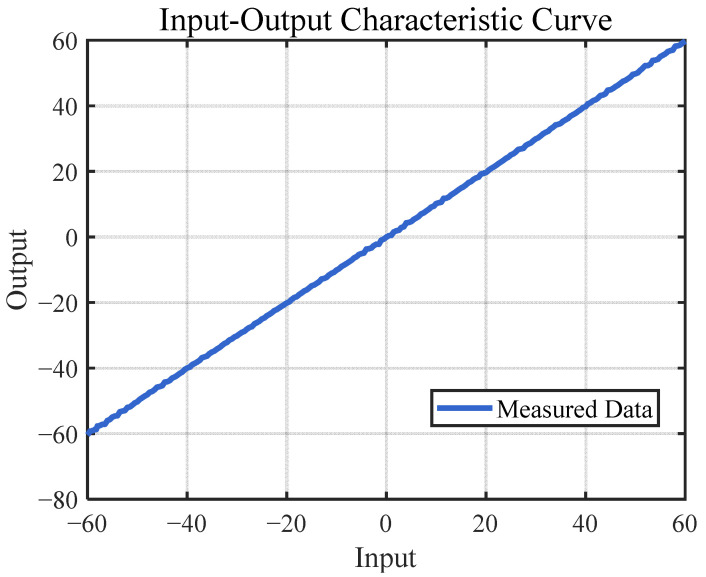
Input-output characteristic curve with a scan step of 1°.

To simulate the RMSE of different DOA methods under the influence of different SNRs, we first set the SNR to 30 dB and the scan resolution to 1°. The DOA results for incident angles ranging from −60° to 60°, covering 121 angles, were simulated and organized into a table, allowing for the construction of the DOA input–output characteristic curve (Figure 11) with a calculated RMSE of 0.44°. Using the same method, the RMSE for a scan step of 0.1° was measured to be 0.25°. Table 2 summarizes the simulation results and performance of the proposed digital beamformer.

#### 4.2.3. RMSE Performance of Different DOA Methods

**Figure 12:**
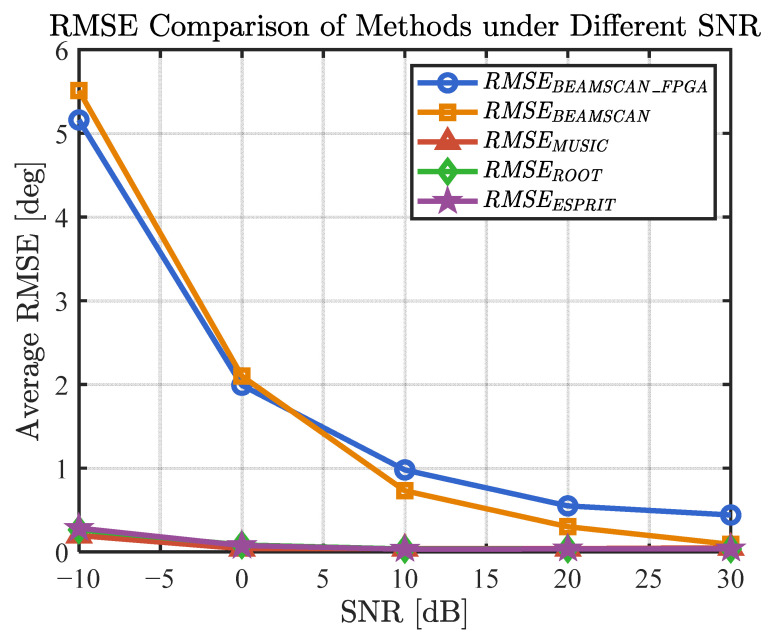
RMSE of different DOA methods under the influence of different SNRs.

Figure 12 compares the RMSE performance of different DOA methods under varying SNR conditions. In low-SNR scenarios (SNR = 0 dB), the beamforming DOA has poor interference resistance, with an RMSE > 2°. In typical application scenarios (SNR = 10 dB), the RMSE of the beamforming DOA is <1°, and it can be used normally. In high-SNR scenarios (SNR = 30 dB), due to the limitation of quantization errors in the FPGA-implemented beamforming method, the RMSE is approximately 0.44°. In MATLAB simulations, where quantization errors are not considered, the best achievable RMSE for beamforming DOA is approximately 0.09°, but it is still higher than that of MUSIC (0.05°), ROOTMUSIC (0.04°), and ESPRIT (0.04°).

In Table 2, the proposed DOA (direction-of-arrival) estimation algorithm in this paper is compared in detail with techniques from other papers. In terms of algorithm type, this paper employs the beam-scanning algorithm, which achieves a good balance between implementation complexity and performance compared to the full-hardware implementation in [22], the Bartlett algorithm in [40], the MUSIC algorithm in [16], and the Cholesky algorithm in [41]. Regarding RMS error, the proposed design achieves an error of 0.25°, which is comparable to other high-precision algorithms ([22]: 0.26°, [16]: 0.49°, [41]: 0.39°), demonstrating its significant advantage in direction estimation accuracy. However, the proposed algorithm requires a higher SNR (30 dB) to achieve the same DOA accuracy, indicating weaker noise suppression capability. In terms of the number of signal sources, the proposed design supports one or two signal sources, which is comparable to [42] and [41] and superior to [22] and [16] (which support only one signal source), showing its ability to maintain high estimation accuracy even in the presence of interfering signals. In terms of processing time, the shortest processing time of the proposed design is 3.62 µs, demonstrating excellent real-time processing capability. In summary, the proposed DOA estimation algorithm exhibits significant advantages in RMS error and processing speed. Although its noise suppression capability is relatively weak, the proposed design performs exceptionally well in direction estimation accuracy and multi-signal source adaptability, making it suitable for real-time DOA estimation applications.

#### 4.2.4. Comparison with Other Designs

The resource, clock frequency, and system configuration comparison of the proposed digital beamformer with other designs is summarized in Table 3.

The digital beamformer (DBF) proposed in this paper is reconfigurable, supporting both DOA mode and conventional beamforming mode. By comparing the resource usage between the reconfigurable architecture and the conventional architecture, it is found that the differences in LUTs, FFs, BRAMs, and DSPs are minimal (LUTs difference of approximately 2.9%, FFs difference of approximately 2.2%, DSPs difference of approximately 11.5%, and no difference in BRAMs). This indicates that the primary resource consumption is concentrated in the multi-channel DDC and complex multiplication sections, while the additional overhead introduced by the DOA mode is small. Compared to the other literature, the proposed design demonstrates superior resource efficiency. This high efficiency makes the proposed design particularly suitable for resource-constrained application scenarios, such as embedded systems or low-cost FPGAs. Additionally, the proposed design shows good applicability in medium-scale application scenarios (eight-channel ULA), supports single-beam operation, and simplifies the model by idealizing the assumptions of the antenna and RF front-end. Compared to the multi-beam design in [42] and the high-complexity designs in [43], the proposed design exhibits significant advantages in resource utilization and computational efficiency, while achieving multi-functional support through reconfigurability. In summary, the proposed design demonstrates outstanding performance in resource efficiency, reconfigurability, and applicability, providing an efficient and flexible solution for the FPGA implementation of digital beamformers.

## 5. Conclusions

In summary, the proposed reconfigurable digital beamformer exhibits significant advantages in direction estimation accuracy, real-time processing capability, and resource efficiency. Simulation results demonstrate that the beamformer can complete DOA estimation with an error of less than 1° within microsecond-level delay under an input signal-to-noise ratio (SNR) of 10 dB, showcasing its real-time processing capability. Although its noise suppression capability is relatively weaker compared to the high-resolution DOA estimation methods, the design performs exceptionally well in multi-signal adaptability and computational efficiency, making it a promising solution for real-time DOA estimation applications. Future work could focus on further optimizing noise suppression and extending the design to support larger arrays and more complex scenarios.

## Figures and Tables

**Table 1:** DOA results when the scanning step is 0.1°.

$\theta_{0}$	SNR = 0 dB	SNR = 10 dB	SNR = 20 dB	SNR = 30 dB
0°	−1.1°	−0.1°	0.6°	0°
−30°	−29.8°	−30.5°	−30.5°	−29.8°
60°	64°	59.7°	59.9°	59.1°

**Table 2:** Comparison of the proposed DOA estimation algorithm with other techniques from the literature.

Specifications	This Paper		[22]	[40]	[16]	[41]
	Step = 0.01°	Step = 0.1°				
Algorithm	Beam Scanning		Full Hardware	Bartlett	MUSIC	Cholesky
RMS error	0.25°	0.44°	0.26°	-	0.49°	0.35°
SNR (dB)	30 8 Kintex-7 1/2		31.19	-	4.37–30	0
Array size			4	4	6	4
Device			-	Cyclone IV	XC7Z020	Virtex-5
Signal Source			1	2	1	2
Excessing Time (μs)	36.02	3.62	-	0.804	2.72	3.08

**Table 3:** Resource, clock frequency, and system configuration comparison of the proposed digital beamformer with other techniques.

Resources	This Paper		[30]	[42]	[43]
	Reconfigurable	Conventional			
LUTs	18,587	18,059	64,327	130,057	394,868
FF	18,633	18,225	52,978	157,746	718,801
BRAM	16	16	93	320	167,490
DSPs	52	46	773	773	1045
Channels	8 ULA	8 ULA	4 ULA	128 ULA	8 × 24 planar
Beams	1	1	1	28	1
Center Frequency	5.5 MHz	5.5 MHz	30 MHz	2.5 MHz	-
Clock	100 MHz	100 MHz	200 MHz	40 MHz	-

## Data Availability

The data that support the findings of this study are available from the corresponding author upon reasonable request.

## Abbreviations

The following abbreviations are used in this manuscript:

DOA	Direction of Arrival
MIMO	Multiple Input Multiple Output
DBF	Digital Beamforming
ULA	Uniform Linear Array
CORDIC	Coordinate Rotation Digital Computer
NCO	Numerically Controlled Oscillator
DDC	Digital Down Converter
CIC	Cascaded Integrator-Comb Filter
ADC	Analog-to-Digital Converter
AF	Array Factor
SNR	Signal-to-Noise Ratio
SIR	Signal-to-Interference Ratio
RMSE	Root Mean Square Error
LMS	Least Mean Squares
FFT	Fast Fourier Transform
MUSIC	Multiple Signal Classification
ESPRIT	Estimation of Signal Parameters via Rotational Invariance Techniques
ROOTMUSIC	Root Multiple Signal Classification
BRAM	Block RAM
CWM	Complex Weight Multiplication
FPGA	Field-Programmable Gate Array
HPBW	Half-Power Beamwidth
FNBW	First Null Beamwidth
FSM	Finite State Machine
RF	Radio Frequency
IF	Intermediate Frequency
